# Behaviour regulation and the role of mental health in non-alcoholic fatty liver disease

**DOI:** 10.1186/s12876-023-02941-x

**Published:** 2023-09-12

**Authors:** E. Asquith, K. Bould, J. C. Catling, E. J. Day, A. Holt

**Affiliations:** 1 School of Psychology, University of Birmingham, Edgbaston, Birmingham, B15 2TT UK; 2 Liver Transplant Unit, Queen Elizabeth Hospital, Birmingham, UK

**Keywords:** NAFLD, Personality disorder, Cognitive restraint, Uncontrolled eating, Anxiety, Depression, Liver disease, Mental health, Locus of control

## Abstract

**Background and aims:**

Non-alcoholic fatty liver disease (NAFLD) has become the most common cause of chronic liver disease in wealthy societies, and is responsible for a significant rise in liver morbidity and mortality. Current treatments prioritise lifestyle interventions, predominantly diet and exercise management, but patients frequently fail to make the necessary behavioural adjustments. The current study seeks to identify those factors which influence patients’ behaviour with respect to adherence to treatment regimes.

**Methods:**

Novel areas of interest were investigated; locus of control, behavioural regulation and a range of mental health measures, due to their links to either poor lifestyle choices or abnormal eating as identified in previous literature. Data was gathered using self-report questionnaires, from 96 participants, who were split into three groups, NAFLD patients, non-NAFLD liver disease patients and healthy controls

**Results:**

Data was analysed using a MANOVA, and followed up with a Tukey post-hoc test. Three factors were found to be significant by group; cognitive restraint, uncontrolled eating and SAPAS score (a measure of personality disorders). An association between personality disorders and NAFLD was identified.

**Conclusion:**

It is suggested that NAFLD patients are screened for personality disorders and, if identified, treated prior to the commencement of diet and exercise management.

## Background

Non-alcoholic fatty liver disease (NAFLD) is the most common chronic liver disease in the Western World [1], and in 2011 NAFLD was reported to be prevalent in up to 25% of the global adult population [2]. NAFLD is a metabolic disorder characterised by the presence of lipid droplets in the liver with the absence of excessive alcohol consumption. Fat accumulates in the liver (in excess of 5% is required for clinical diagnosis), and inflammation and fibrosis occurs [3]. A spectrum of liver pathologies are encompassed within NAFLD, ranging from steatosis to non-alcoholic steatohepatitis (NASH), a progressive disorder which can progress to cirrhosis [4].

NAFLD is a global health problem and a multifaceted disease, with the main risk factors being obesity and insulin resistance [4]. Because of the close relationship with obesity, NAFLD’s prevalence will continue to constitute a major public health challenge as western societies battle with rising rates of obesity-related disease [5]. Standard-of-care treatment for NAFLD is highly dependent on achieving a behavioural change specifically with respect to food choices and weight loss achieved through calorie restriction and increased exercise [6]. However, not all patients take the necessary actions to prevent the disease developing. In one seminal study from Cuba which investigated lifestyle modification in patients with NAFLD only 30% (52/293) of patients enrolled achieved significant weight loss over 52 weeks of intervention. This is despite the fact that no disease progression was seen in the groups who achieved > 7% weight loss from baseline [7]. Food choices and a sedentary lifestyle appear to be key determinants of NAFLD prevalence. One study of 171 biopsy proven NAFLD patients in Japan showed that poor food choices and a decrease in exercise were strongly associated with the presence of NAFLD [8].

Although simple measures such as quantitative and qualitative changes in diet and increased exercise have been shown to prevent disease progression in NAFLD it is often difficult to persuade patients to follow dietary and exercise programmes. NAFLD patients are aware of the beneficial effects of lifestyle modifications, yet frequently lack an ability to make the necessary changes; sufferers’ adherence to an improved lifestyle is poor [9]. Even amongst patients transplanted for NAFLD Dureja et al. [10] found that almost 40% of patients showed signs of disease recurrence within five years of liver transplantation. These findings suggest an urgent need to examine attitudes towards diet and exercise in order to understand motivational pathways that will allow more effective treatment of NAFLD, and prevent disease recurrence after liver transplantation.

One factor that determines attitudes towards, (and maintenance of), weight loss is an individual’s locus of control (LoC). LoC can be defined as how much control one perceives to have over one’s life events, and is split into internal and external components [11]. Those with an internal LoC perceive life events to be a consequence of their own actions and hold the view that they can control their future. In relation to weight loss, those with high internal LoC may see weight loss as under their control and thus are more likely to be successful in losing weight [11]. Nir and Neumann [12] examined LoC as a predictor for long-term maintenance of weight loss. Over a 10-week weight-loss programme they found that individuals with higher internal LoC had less weight regain, suggesting that higher levels of internal LoC improved personal responsibility for an individual’s actions and maintained changes to their health-related behaviour in the longer-term. This is an important observation as NAFLD sufferers, rather like individuals with substance abuse disorders, may have increased external LoC, such that they see life events as not being under their control and therefore they struggle to implement and maintain the necessary changes to their diet and exercise regime that prevent disease progression into the more serious, irreversible stages of the disease.

Mental health also influences attitudes towards diet and exercise. Specifically. a number of personality disorders (PDs) (e.g. Antisocial Personality Disorder (ASPD)) are associated with higher impulsivity scores compared to healthy controls [13]. We know that people with personality disorder often have problems with interpersonal relationships but often attribute them wrongly to others. No clear threshold exists between types and degrees of personality dysfunction and its pathology is best classified by a single dimension, ranging from normal personality at one extreme through to severe personality disorder at the other. We also know that comorbidity with other mental disorders is common, and the presence of personality disorder often has a negative effect on course and treatment outcome [14]. A recent systematic review of personality disorders and obesity by Gerlach et al., [15] found that adults with any personality disorder had a higher risk of obesity, and in the female general population there is an association between avoidant or antisocial PD and severe obesity. Patients with PD are less likely to succeed with conservative weight-loss treatment programmes for obesity and it is likely that treatment programmes will need to be adapted to take the presence of PD into account. In support of this, Wilson et al. [16] identified non-planning impulsivity in borderline personality disorder (BPD) where individuals have difficulty in planning and have a poor understanding of the consequences of their actions. This relates to NAFLD treatment and sufferers’ difficulty planning and changing diet and exercise habits. Impulsivity has also been linked to overeating; Guerrieri and colleagues [17] manipulated impulsivity and found that it was causally linked with overeating. Maintenance of a healthy diet is a key aspect of NAFLD treatment, and impulsive tendencies resulting in overeating may be a factor that can predict whether or not an individual will have success in their dieting [18]. In sum this research suggests that personality disorders may be involved in the inability of some NAFLD patients to recover.

Anxiety and depression have also been linked to obesity with emotional eating significantly positively related to anxiety and depression [19]. This suggests that food is being used as a coping strategy. Additionally, those who are obese are more likely to suffer with depression [20]. Consequently, there is a potential feedback loop, whereby individuals with a mental health problem overeat, leading to NAFLD, which itself can contribute to mental health issues.

The current study is exploratory in nature and aims to identify factors relating to NAFLD prevalence by comparing NAFLD patients to those with a non-NAFLD liver disease and healthy controls. Currently, NAFLD research has been narrowly focussed around outcome e.g. diet and exercise habits, but additional factors need to be identified that could shed light on the inability of some NAFLD patients to make the necessary health-related behavioural changes. Identification of such factors has the long-term potential to improve NAFLD treatment success.

## Methods

### Participants

A sample of 96 participants (53 females), between the ages of 19 and 78, was split into three groups; 34 NAFLD patients (16 females), 25 with non-NAFLD liver disease such as hepatitis B or primary biliary cirrhosis (15 females) and 37 healthy controls ( 22 females). A priori sample size calculations using G-Power [21] using a power value 0f 0.8 and expected medium effect sizes gave a suggested total sample size of 66. Opportunity sampling was used to recruit participants; NAFLD and non-NAFLD liver disease patients were recruited from the tertiary NAFLD research clinics and chronic hepatitis B and PBC clinics at QEHB. Importantly, all patients completed an assessment to preclude any form of alcohol use disorder. Healthy controls were either hospital staff, researchers or members of the public. The mean age of the sample was 53.60 (SD = 11.81), and the means of the three groups were 54.41 years for the NAFLD patients, 56.36 for the non-NAFLD liver disease patients, and 50.97 years for the healthy controls.

### Materials

All participants completed six questionnaires: a demographic questionnaire including variables that may relate to NAFLD prevalence such as ethnicity and education, and five further questionnaires each investigating an individual factor.

### Revised three factor eating questionnaire

Eating behaviours of participants were gauged using Karlsson et al.’s [22] Revised Three Factor Eating Questionnaire (TFEQ-R18). This consisted of 18 items: 13 statements rated on a 4-point Likert scale from 1 (definitely false) to 4 (definitely true) for example. “When I feel blue, I often overeat”; four questions answered on a variety of 4-point Likert scales; and one scale rating from 1 (no restraint) to 8 (total restraint). The TFEQ-R18 consists of three subscales; cognitive restraint eating, uncontrolled eating and emotional eating, a high score indicated a high level on these subscales. The Cronbach’s alpha reliability was above 0.7 [21], signifying acceptable internal consistency.

### Behavioural Regulation in Exercise Questionnaire – 2 (BREQ-2) [23]

This was implemented to assess exercise habits. Nineteen statements e.g. were rated on a 5-point Likert scale (0 = not true for me, 4 = very true for me) e.g. “I enjoy my exercise sessions”,. Scores on five subscales were generated; amotivation, external regulation, introjected regulation, identified regulation and intrinsic regulation. A high score indicated a high level on these subscales. The mean score of each subscale was used to determine the overall Relative Autonomy Index (RAI) i.e. the degree to which respondents feel self-determined. All subscales within the BREQ-2 were found to have a Cronbach’s alpha reliability coefficient between 0.73 and 0.86 [23].

### Locus of control questionnaire

Rotter’s Locus of Control (LoC) Questionnaire [24] was administered to assess how much control individuals feel they have over life events. This consisted of 29 pairs of statements and participants had to choose which they agreed with most (forced-choice test) e.g. a) “There are certain people who are just no good”, or b) “There is some good in everybody”. A low score indicated a high level of internal LoC. Kuder-Richardson reliability was chosen to assess internal consistency because of the dichotomous-choice nature of this questionnaire, and r values ranging between 0.69 and 0.78 were considered acceptable.

### Hospital anxiety and depression scale

Psychiatric co-morbidity was assessed using the Hospital Anxiety and Depression scale (HADS) [25], which measured the degree of anxiety and depression. Participants were presented with 14 statements (for example “I feel miserable and sad”) to respond to on a 4-point Likert scale from 0 (no, not at all) to 3 (yes, definitely). The internal consistency reliability, assessed with ordinal alpha, is 0.92 for HADS Anxiety and 0.88 for HADS Depression. The corresponding internal consistency measured with traditional Cronbach’s alpha is 0.87 and 0.81 respectively [26].

### Standard assessment of personality-abbreviated scale

PDs were identified using the Standard Assessment of Personality-Abbreviated Scale (SAPAS;) [27], consisting of eight questions with yes/no answers, for example “Are you normally a worrier?”. Moran et al. showed that a score of 3 on this screening interview correctly identified the presence of DSM-IV personality disorder in 90% of participants, with a sensitivity and specificity of 0.94 and 0.85 respectively.

## Procedure

The research was conducted in the out-patient liver clinic at the QEHB. QEHB consultants identified suitable NAFLD and non-NAFLD liver disease patients and directed them to the researchers for recruitment. All patients had received a diagnosis of NAFLD based on biopsy and/or biochemical, imaging and clinical assessment by a Transplant Hepatologist before inclusion in the study. Other patients were recruited directly from the hepatitis B clinic or autoimmune liver disease clinics. Healthy controls were recruited directly by the researchers. Researchers read out the six questionnaires to all participants, who answered verbally.

### Ethics

Participants provided informed consent prior to the study and were given a full debrief form with researchers’ contact information in case of queries. The right to withdraw was offered throughout the study and all data were kept confidential.

### Statistical analysis

A multivariate analysis of variance (MANOVA) was undertaken to determine the effect of group (NAFLD vs. non-NAFLD liver disease vs. healthy control) on eating habits, behaviour regulation, Locus of control, anxiety and depression. Follow-up univariate ANOVAs were used to assess for significant differences between the individuals in the different groups. Tukey post-hoc tests were conducted to identify where the group differences occurred.

## Results

From the MANOVA analysis, the differences between the groups on the combined dependent variables was statistically significant, F(26, 160) = 2.178, *p* = 0.002; Wilks' Λ = 0.546. Follow-up univariate ANOVAs identified statistically significant differences between the individuals in the different groups on scores for the SAPAS scale (F(2, 92) = 7.553, *p* = 0.001), uncontrolled eating on the TFEQ-R18 scale (F(2, 92) = 5.697, *p* = 0.005) and cognitive restraint on the TFEQ-R18 scale (F(2.92) = 4.413, *p* = 0.015). No other factors showered significant differences.

**Fig. 1:**
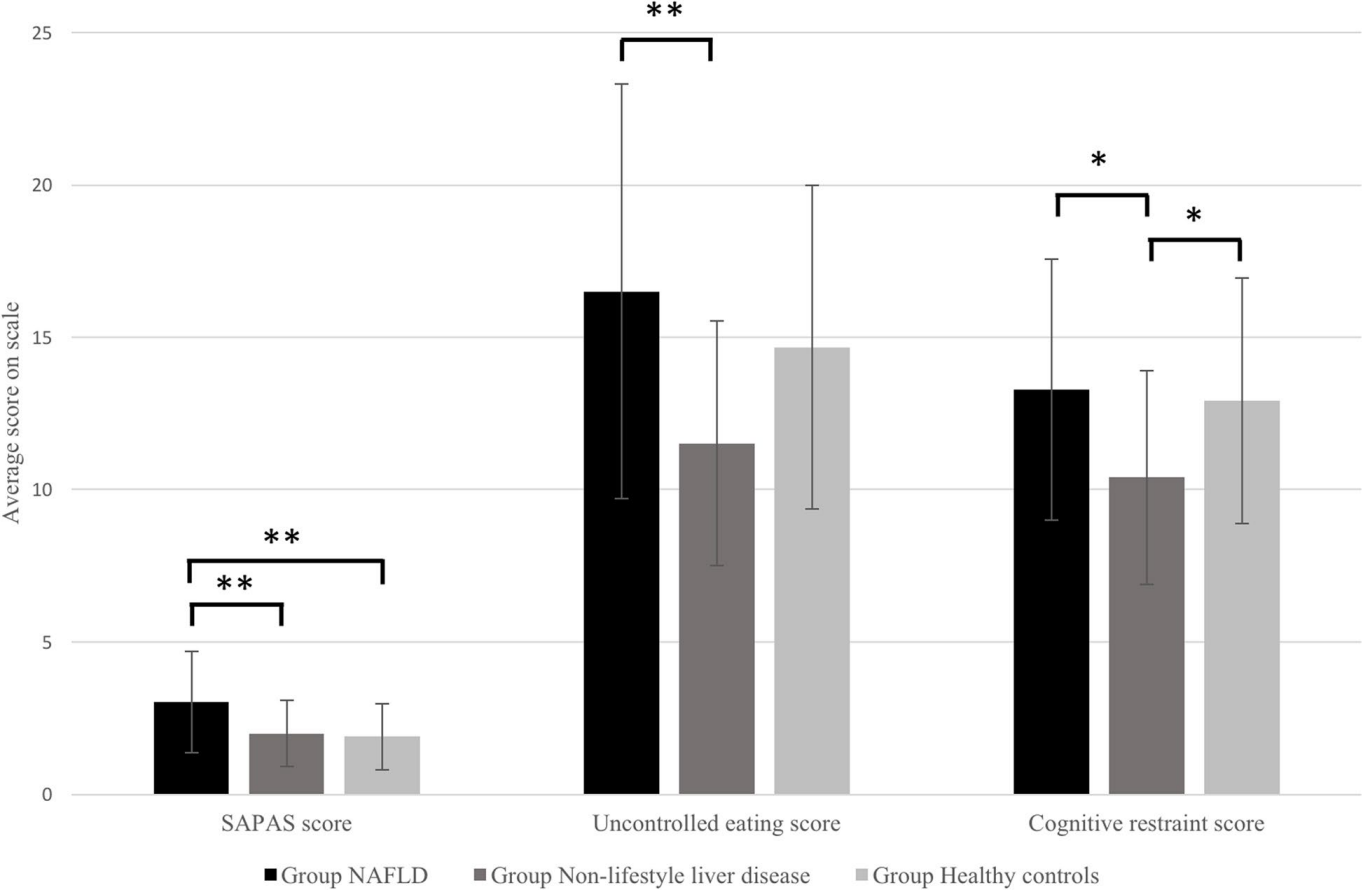
Differences in average scores for the factors on which scores were significantly different between the three groups (*p < 0.05, **p < 0.01; error bars denote the standard deviation)

The Tukey post-hoc tests showed that on the SAPAS scale, NAFLD patients had statistically significantly higher mean scores than both non-NAFLD liver disease patients (*p* = 0.011) and healthy controls (*p* < 0.001). In terms of uncontrolled eating, NAFLD patients had statistically significantly higher mean scores than non-NAFLD liver disease patients (*p* = 0.003). In terms of cognitive restraint, NAFLD patients had statistically significantly higher mean scores than non-NAFLD liver disease patients (*p* = 0.017). Non-NAFLD liver disease patients had statistically significantly lower mean scores than healthy controls (*p* = 0.042). No other factors showered statistically significant differences. Mean scores for these three significant factors are shown in Table 1, and graphical representation of differences between the groups is shown in Fig. 1.

**Table 1:** Mean scores for each factor on which scores were significantly different between the three groups (standard deviation in parentheses)

		Scale	
Group	SAPAS	TFEQ-R18 Uncontrolled eating	TFEQ-R18 Cognitive restraint
NAFLD patients (*N* = 34)	3.03 (1.66)	16.50 (6.80)	13.29 (4.29)
Non-NAFLD liver disease patients (*N* = 25)	2.00 (1.08)	11.52 (4.02)	10.40 (3.50)
Healthy controls (*N* = 37)	1.89 (1.09)	14.67 (5.31)	11.52 (4.02)

Table 2 shows the percentage of individuals in each group that met the recommended clinical cut-off score of either three or four for diagnosis of a PD according to the SAPAS scale, depending on level of caution as cited in Moran et al. (2003) [27].

**Table 2:** Percentage of participants within each group that met or surpassed the recommended clinical cut-off score of 3 or 4 on the SAPAS scale

Group	Score	Score 4
NAFLD patients (*N* = 34)	64.71%	29.41%
Non-lifestyle liver disease patients (*N* = 25)	36.00%	8.00
Healthy controls (*N* = 37)	22.22%	8.33%

## Discussion

NAFLD patients were found to be significantly more likely than non-NAFLD liver disease patients and age-matched healthy controls to suffer with a Personality Disorder (PD). Additionally, patients with NAFLD were more likely to exhibit uncontrolled eating disorders than non-NAFLD liver disease patients. However, it was also found that non-NAFLD liver disease patients had less cognitive restraint of food intake than NALFD patients and healthy controls. This may reflect the fact that these patients were often encouraged to boost their protein and calorie intake to reverse the nutritional declines commonly seen in chronic liver disease. Moreover it highlights that whilst NAFLD patients are aware of what they need to do, they frequently exhibit uncontrolled eating behaviours which might be indicative of a disconnect between knowing the right behaviour and performing the behavioural adjustment. No other assessed factors were found to have a statistically significant difference between NAFLD patients and the other two groups.

To the researchers’ knowledge, this is the first study to link NAFLD to PD. Table 2 highlights the clinical relevance of this finding; 64.71% of the NAFLD patients met or surpassed the SAPAS cut-off score of three, indicating a 90% chance of having a PD (Moran et al., 2003) [27]. In comparison, far fewer (36%) non-NAFLD liver disease patients met this cut-off. Of the healthy controls, 22.22% were found to have a PD as assessed via SAPAS, which is high in comparison to studies that have assessed PD prevalence in the general population. For example, Oltmanns, Rodrigues, Weinstein and Gleason [28] found the prevalence to be around 7% in a community sample according to Structured Interview for DSM-IV Personality (SIDP-IV), a self-report questionnaire and an informant questionnaire. However, in the original SAPAS paper (Moran et al., 2003) [27], they suggest a higher prevalence in the general population of between 10–20%, which would be relatively consistent with the 22.22% obtained for healthy controls in this study. Even if we only consider those patients that met or exceeded a threshold score of four (most authorities would suggest that a score of three is the optimal diagnostic threshold in terms of sensitivity and specificity [27]) the difference remained clinically significant; 29.41% of the NAFLD patients scored at least four, compared to only 8.00% of the non-NAFLD liver disease patients and 8.33% of healthy controls. With the use of either cut-off score, NAFLD patients were around three times more likely to have a PD than healthy controls.

The observation of a significantly increased prevalence of PD in NAFLD patients is particularly striking because the higher SAPAS scores found amongst NAFLD patients compared to healthy controls were not found in non-NAFLD liver disease patients. This signifies that it is not an issue associated with all liver disease, but specifically related to those with NAFLD. Importantly, it appears not to be a general mental health issue, as neither anxiety nor depression were found to be significantly different between the groups, despite both of these psychiatric disorders often being co-morbid with chronic liver disease [29, 30].

The link between PD and NAFLD could be mediated by impulsivity. As stated earlier, both Swann et al. [13] and Wilson et al. [16] found impulsivity to be linked to numerous PDs, which could explain the results obtained in the present study. This leads to a potential hypothesis as to why some NAFLD patients are unable to recover. The impulsivity in those with PDs is preventing those who also suffer with NAFLD from improving their diet, as impulsive tendencies result in overeating [17, 18]. Borderline Personality Disorder (BPD) is a complex and severe mental disorder characterised by emotional, social, self-image and impulse control dysregulation [31]. BPD sufferers exhibit high risk and impulsive behaviours across many domains [32], it may be that those who also suffer with NAFLD express this higher risk behaviour in their inability to maintain a prescribed diet, or difficulty controlling impulses to consume the food types they need to avoid. Additionally, individuals with BPD have been found to have difficulty with decision making and are less able to use feedback information. For example, when BPD patients completed a modified version of the Iowa Gambling Task and results were compared to healthy controls, Schuermann et al. [33] found that patients with BPD made higher risk choices and were unable to improve their performance with the positive and negative information provided, highlighting their inability to adapt their behaviour to avoid disadvantageous situations. This suggests that individuals with BPD (or other PDs) and NAFLD may have a higher appetite for risk and fail to process information provided by clinicians designed to modify behaviours and food choice despite having been made aware of the consequences of not adapting their lifestyle. However, this proposed link has two caveats. First, the directionality of the relationship is unknown, as mental health problems can result from physical health issues [33]. Secondly, the relationship between NAFLD and PDs has only been shown within a cross-sectional methodology at this stage and longitudinal research would be required to determine causality.

Significantly higher levels of uncontrolled eating were found in patients with NAFLD when compared with patients with non-NAFLD liver disease, and higher (but not significantly higher) levels than in healthy controls. If further research is conducted with a larger sample the difference between NAFLD patients and healthy controls would be expected to become significant, consistent with previous literature. Individuals who consume more calories, often because of an inability to control food intake, have been found to have higher increases in levels of liver fat [7, 34] and NAFLD prevalence has been found to be higher within groups of obese individuals compared to those with a healthy weight [35, 36]. Moreover, obesity has been found to be partly the result of uncontrolled eating [37], providing a clear causal link between the present findings of high levels of uncontrolled eating within the NAFLD group.

The higher cognitive restraint of food intake scores found amongst patients with NAFLD may seem contradictory to previous research, but is not surprising; NAFLD patients were attending consultations with a dietician in which they were advised on which food groups to avoid. Additionally, the significantly lower cognitive restraint scores amongst non-NAFLD liver disease patients than that of both NAFLD patients and healthy controls could be explained by the reduced appetite of Hepatitis B sufferers [38], who made up the majority of participants in this group. Subsequently, these individuals do not have a desire to eat in large quantities and therefore do not need to restrict their intake.Nir and Neumann [12] found LoC to be a predictor of long-term weight loss, suggesting a potential link between attitudes towards diet and exercise amongst NAFLD patients and LoC. However, the present study did not find a significant difference in LoC scores between NAFLD patients and non-NAFLD liver disease patients or healthy controls. Lack of exercise was also not found to be significant, despite the known relationship between NAFLD prevalence and a sedentary lifestyle [9], but the NAFLD patients in this study were recruited from a tertiary NAFLD research clinic where they were repeatedly advised to increase physical activity level by the medical team: It would be interesting to repeat these questionnaires in a non-tertiary liver clinic where exercise and dietary objectives are less vigorously endorsed.

The greater prevalence of PD in NAFLD patients has implications for treatment. It may be more effective to treat an individual’s underlying PD before the NAFLD is treated, as the mental health issues could influence attitudes towards diet and exercise and therefore preventing the necessary behaviour changes. Currently, the main treatment for PD is CBT [39] or psychotherapy [40], and there could be benefits in implementing these therapies prior to patients attempting lifestyle changes.

The use of self-report measures is a limitation in this study as they might introduce a social desirability bias [41], whereby participants may answer in a way that presents themselves as more favourable to the interviewer. This risk of bias is heightened amongst the clinical group patients (NAFLD and non-NAFLD liver disease patients) as they completed the study immediately after a clinical consultation. However, the finding of higher rates of PDs among NAFLD patients suggests that this bias is unlikely to have impacted the results. Nonetheless, future research should aim to assess whether the findings can be replicated when the study is conducted in a neutral environment with follow up sessions and input from informants close to the participants.

Further significant limitations of the current study are related to the data analysis and methodology—First, the directionality of the relationship between mental health problems and NAFLD cannot be conclusively attested to as we know that mental health problems can result from physical health issues and vice versa. Secondly, the relationship between NAFLD and PDs has only been shown within a cross-sectional methodology at this stage and strictly speaking longitudinal research would be required to determine causality. Finally, the current study did not assess for BMI/obesity levels throughout the three groups, and this could potentially have acted as a confounding variables within the performed statistical analysis.

It should be noted that the questionnaire chosen as a measure of PD presence (SAPAS) did not specify which PD was present in the participants. This highlights the importance of following up on these novel observations and investigating whether the findings reflect particular PDs, as this would facilitate adaptation of education and therapeutic material, and modification of educational techniques interventions. Finally, attitudes towards diet and exercise of NAFLD patients could be tested after they have completed PD treatment schemes to see if there is a causal relationship.

## Conclusion

The successful lifestyle adjustments indicated for the treatment of NAFLD are simple and proven, whereby the patient must improve their diet and increase exercise, and yet patients with NAFLD are remarkably resistant to implementing and maintain effective behavioural change. This study has identified a previously unreported association between PDs and NAFLD which will explain many of these observations and offers a new therapeutic insight into the behavioural and volitional mindset of the patient with NALFD. In conclusion, we would suggest that that NAFLD patients are screened for PDs and, if identified, treated prior to implementation of diet and exercise management strategies.

## Data Availability

All data has been uploaded as an additional materials file.

## Abbreviations

NAFLD: Non Alcoholic Liver Disease
LoC: Locus of Control
PD: Personal Disorder
QEHB: Queen Elizabeth Hospital Birmingham
TFEQ-R18: Revised Three Factor Eating Questionnaire
BREQ-2: The Behavioural Regulation in Exercise Questionnaire– 2
HADs: Hospital Anxiety and Depression scale
SAPAS: Standard Assessment of Personality-Abbreviated Scale
